# Quantitative analysis of orthopedic metal artefact reduction in 64-slice computed tomography scans in large head metal-on-metal total hip replacement, a phantom study

**DOI:** 10.1186/s40064-016-2006-y

**Published:** 2016-04-02

**Authors:** Martijn F. Boomsma, Niek Warringa, Mireille A. Edens, Dirk Mueller, Harmen B. Ettema, Cees C. P. M. Verheyen, Mario Maas

**Affiliations:** Department of Radiology, Isala Hospital, Zwolle, The Netherlands; Department of Innovation and Science, Isala Hospital, Zwolle, The Netherlands; Philips GmbH, Hamburg, Germany; Department of Orthopedics, Isala Hospital, Zwolle, The Netherlands; Department of Radiology, AMC, Amsterdam, The Netherlands

**Keywords:** CT, MoM, THA, Metal artefacts, O-MAR

## Abstract

**Purpose:**

Quantification of the effect of O-MAR on decreasing metal artefacts caused by large head metal on metal total hip arthroplasty (MoM THA) in a dedicated phantom setup of the hip.

**Background:**

Pathological reactions of the hip capsule on Computed tomography (CT) can be difficult to diagnose due to different metal artefacts. The O-MAR algorithm deploys an iterative loop where the metal sinogram is identified, extracted, and subsequently serves as a mask to correct the measured sinogram. Main goal of this study is to quantify the ability of the O-MAR technique to correct deviation in medullary bone attenuation caused by streak artefacts from the large-head MoM THA embedded in a phantom. Secondary goal is to evaluate the influence of O-MAR on CNR.

**Methods:**

The phantom was designed as a Perspex box (PMMA) containing water and a supplementary MOM THA surrounded by Perspex columns comprising calibrated calcium pellets. Each column contains 200 mg of hydroxyapatite/calcium carbonate to simulate healthy bone tissue. Scans were obtained with and without a MoM THA at different dose levels. Different reconstructions were made with filter A, iDose^4^ level 5 and with and without O-MAR. The scans without the prosthesis were used as the baseline. Information about the attenuation in Hounsfield units, image noise in standard deviation within the ROI’s were extracted and the CNR was calculated.

**Results:**

Pellet L0 and R0 (proximal of the MoM THA) were defined as reference, lacking any disturbance by metal artefacts; L5, L6 and L8 were respectively visually categorized as ‘light’ ‘medium’ and ‘heavy disturbance’. Significant improvements in attenuation deviation caused by metal artefact were 43, 68 and 32 %, for respectively pellet L5, L6 and L8 (p < 0.001). Significant CNR improvements were present for L5 and L6 and were respectively 72 and 52 % (p < 0.001). O-MAR showed no improvement on CNR for L8.

**Conclusion:**

This phantom study significantly increases image quality by the use of O-MAR in the presence of metal artefacts by significantly reducing metal artefacts subsequently and increasing CNR on a 64 slice CT system in light and medium disturbance of the image.

## Background

Metal-on-metal total hip artroplasties (MoM THA) were introduced because of their purported advantages above the conventional metal-on-polyethylene articulations (Voleti et al. 2012). Favorable patient satisfaction, lower rates of dislocation, wear and good survival have been reported at medium-term follow-up (Haddad et al. 2011). However, there have been reports of the formation of symptomatic peri-articular masses in some patients, referred to as pseudotumours (Ollivere et al. 2009). The exact incidence of these pseudotumours is unknown, seems to differ between type of MoM prosthesis. These so-called pseudotumours or pathological capsular reactions of the hip after MoM THA are seen in different frequencies depending on prosthesis type and population (Bisschop et al. 2013; Bosker et al. 2012, 2015). It is generally accepted that revision surgery is warranted to halt the process of pseudotumour formation (Daniel et al. 2012). Radiological imaging studies are used in screening protocols in symptomatic patients to identify those patients that are candidates for revision surgery (Boomsma et al. 2015). This has led to a dramatic increase in the demand for imaging studies (Robinson et al. 2014).

Various imaging studies are described for diagnosing pseudotumours. Computed tomography (CT) is relatively inexpensive, readily available and very sensitive in illustrating bone defects and has the important advantage that orientation of components can be measured. It exposes patients however to a certain amount of radiation. Pathological capsular reactions can be difficult to diagnose with MR or CT due to different metal artefacts caused by the high atom number of these MoM-prostheses and therefore hide underlying pathological capsular reactions.

Previously a reliable classification system has been established for reporting CT appearances of the hip capsule and pseudo tumours in MoM disease that shows significant association with revision (Boomsma et al. 2015). We investigated several clinical populations in two different hospitals during follow up. By analyzing CT scans made in follow up from these different hospitals we encountered marked quality differences in visual acuity in the various used Philips CT systems (16, 48, 64, 128 and 256 in different generations). We found diagnostic improvement in imaging quality by use of the latest multi-slice systems together with Orthopedic Metal Artifact Reduction (O-MAR, Philips Healthcare, Cleveland, OH, USA) post processing software in our current clinical daily practice.

The O-MAR algorithm deploys an iterative loop where the metal sinogram is identified, extracted, and subsequently serves as a mask to correct the measured sinogram. For more detailed description of O-MAR we refer to “Appendix”.

For the subsequent interpretation of the results from the use of O-MAR it is important to reflect here shortly on the different contributions for image noise and artefacts in CT.

Noise comprises random and structural components. Random noise can be attributed to purely statistical variations inherent in all physical phenomena. More specific for CT: quantum noise and electronic noise e.g. in the A/D conversion. It is possible to characterize the average behavior of the noise by a variety of statistical methods (e.g. histogram analysis and SD) (Newton and Potts 1981).

Pronounced high density variations in the scanned object lead to structural noise, i.e. artefacts, caused beam hardening and photon starvation. As the X-ray beam passes through a dense object such as a metal implant, more low-energy photons are absorbed as compared to high-energy photons, leading to a shift of the X-ray spectrum. Secondly, dense structures, such as metal, attenuate photons to a degree where the photon flux is so low, that the detectors are ‘starved’ for photons. Filtered back-projection (FBP) has been the industry standard for CT image reconstruction for decades. While it is a very fast and fairly robust method, FBP is sub-optimal for undersampled data or for noisy data. When these very high levels of noise are propagated through the reconstruction algorithm, the result is an image with significant artefacts and high degrees of random noise. The resulting artefacts can be seen as streak artefacts, which after rescanning remain at very similar angles throughout the image. These artefacts remain with the use of combination protocols consisting of iterative reconstruction techniques and FBP.

O-MAR has previously shown to be effective in reducing metal artefacts in dental implants, planning radiotherapy with implants and in a phantom (Hilgers et al. 2014; Kidoh et al. 2014; Li et al. 2012; Philips CT Clinical Science, Philips Healthcare USA 2012). This and our personal observation of a positive effect regarding metal artefact reduction have not yet been extensively quantified for MoM THA in a phantom. We decided to quantify the effect of O-MAR on metal artefacts in a phantom setup that resembles the imaging situation of our local clinical population of large head MoM THA. In this way we tried to correlate our aforementioned subjective visual observations with objective quantitative image quality estimates in a phantom study.

The main goal of this study is to quantify metal artefact reduction by O-MAR, caused by streak artefacts from the large head MoM THA in a phantom. The secondary goal is to evaluate the influence of O-MAR on CNR.

## Methods

### Phantom design

**Fig. 1:**
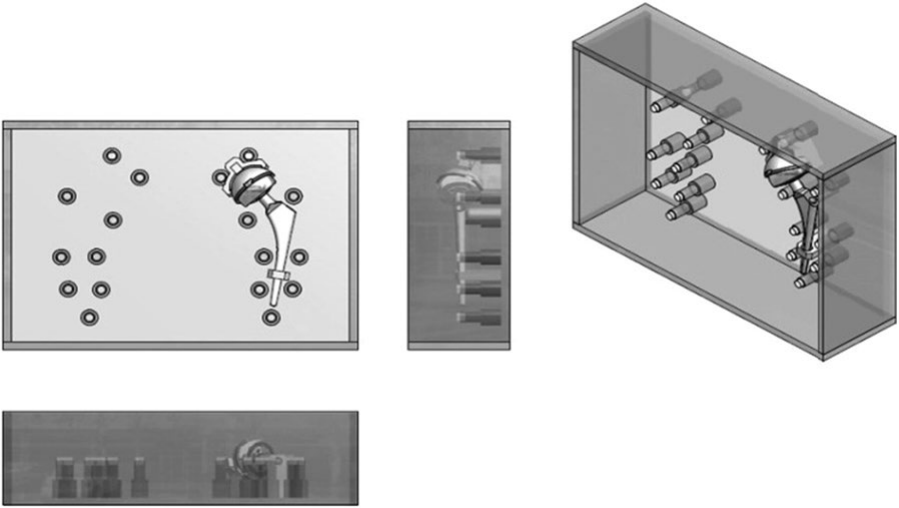
Schematic drawing of the phantom used in this study. Measurements are performed on the calcium pellets embedded in Perspex columns

The phantom walls were made of a polymethyl-methacrylate (Perspex) to form a watertight box open at the top. Inner dimensions of width, length and height were 42 × 29.5 × 13 cm. To simulate heavy patients the box dimensions in the x, y, z plane were designed to corresponded to a Water-Equivalent Diameter (WED) of 39.7 cm. The box held 18 columns as shown in Fig. 1. The columns with calibrated calcium pellets were placed at standard Gruen zones and DeLee and Charnley regions that are used for bone analysis near the stem and at other critical locations such as the assumed acetabulum (DeLee and Charnley 1976; Gruen et al. 1979). Each column contained 200 mg of hydroxyapatite/calcium carbonate (HA/CC, from company QRM, Möhrendorf, Germany) to simulate healthy bone tissue. The pellets were embedded within a polymethyl-methacrylate (Perspex) and were not suspended. Within a tolerance of 0.05 and 0.1 mm in the X, Y and Z-axis the center of the pellets were situated within one plane. The pellets had a height of 1 cm and a diameter of 1 cm. On the left side of the phantom the supplementary placed large head MoM THA prosthesis could be attached by fitting into Perspex holders in the phantom with its main axis falling into the plane defined by the pellets. No additional fixation was needed. The Perspex box was always, with or without the MOM THA completely and equally filled with tap water. The air bubbles that arose around the columns were removed carefully to create a homogenous density. A silicon foil was placed on top of the box before the cover was placed, ensuring that any residual air on the sides was removed (Fig. 1). Prior to scanning on a Philips Brilliance 64 CT-scanner (Philips Healthcare, Cleveland, OH, USA), an air calibration was performed. Additionally a scan was made by which randomly the HU values across the Field of View (FOV) were checked to lie within the tolerance of HU and SD defined by the CT manufacturer in the product specifications. Because of the small amount of scans that were necessary for the study [two X-ray tube voltage (kV_p_) settings, three current–time product (mAs) values and with and without MOM THA prosthesis, leading to 12 scans] the variations in tube temperature were negligible. Scans were repeated by duplicating the static scan settings (Table 1), apart from the variables under investigation. The phantom was placed on the table of the CT scanner and it’s position was checked for displacement after each scan performed. This enabled to create consistent DICOM image data sets, with as little uncontrolled variation as possible.

**Table 1:** Applied scan parameters

Scan parameters	Setting
FOV (reconstructed)	455 mm
Resolution	High resolution
Pitch	0.49
CTDI (120/140 kVp)	
101/150 mAs	9.8 mGy
202/300 mAs	19.6 mGy
405/600 mAs	39.3 mGy
Collimation	64 × 0.625 mm
Collimation speed	0.75 s/rotation
Pixel size	0.44 mm
Matrix	1024 × 1024
Scan length	303 mm
Slice thickness	0.90 mm
Increment	0.45 mm
Dose modulation	Off
Zoom factor	1
SP filter	On
Adaptive filter	On

### Image acquisitions

The phantom was scanned with and without the MoM-hip prosthesis using different scan parameters. The scans were reconstructed with Filter A and O-MAR. Filter A was used in order for later research purposes and for clinical resemblance in reading soft tissues around the hip. With ImageJ, Region of Interest (ROIs) were placed in the calibrated calcium pellets and in the surrounding water at standard Gruen zones and at other particularly critical locations (DeLee and Charnley 1976; Gruen et al. 1979). Information about the attenuation in Hounsfield units and image noise in standard deviation (SD) within the ROIs were extracted and the contrast-to-noise ratio (CNR) was calculated. The scans without the prosthesis were used as the baseline. Effects of O-MAR in conjunction with different scan parameters were investigated based on the different combinations in helical scan mode. The current–time product mAs with a X-ray tube voltage setting of 140 kVp was lowered to create an equal Computed Tomography Dose Index (CTDI) as with the setting of 120 kVp (Table 1). Scans were made with CTDI’s of respectively 9.8, 19.6 and 39.6.

All scans were reconstructed with level 5 of the iterative reconstruction method iDose^4^ (Philips Medical Systems, Cleveland, OH, USA). This was the strongest combination of iterative reconstruction with FBP.

All the used parameters in this phantom study were based on patient protocols to mimic the clinical environment as much as possible, with adequate dose (ALARA).

### Quantitative measurements

**Fig. 2:**
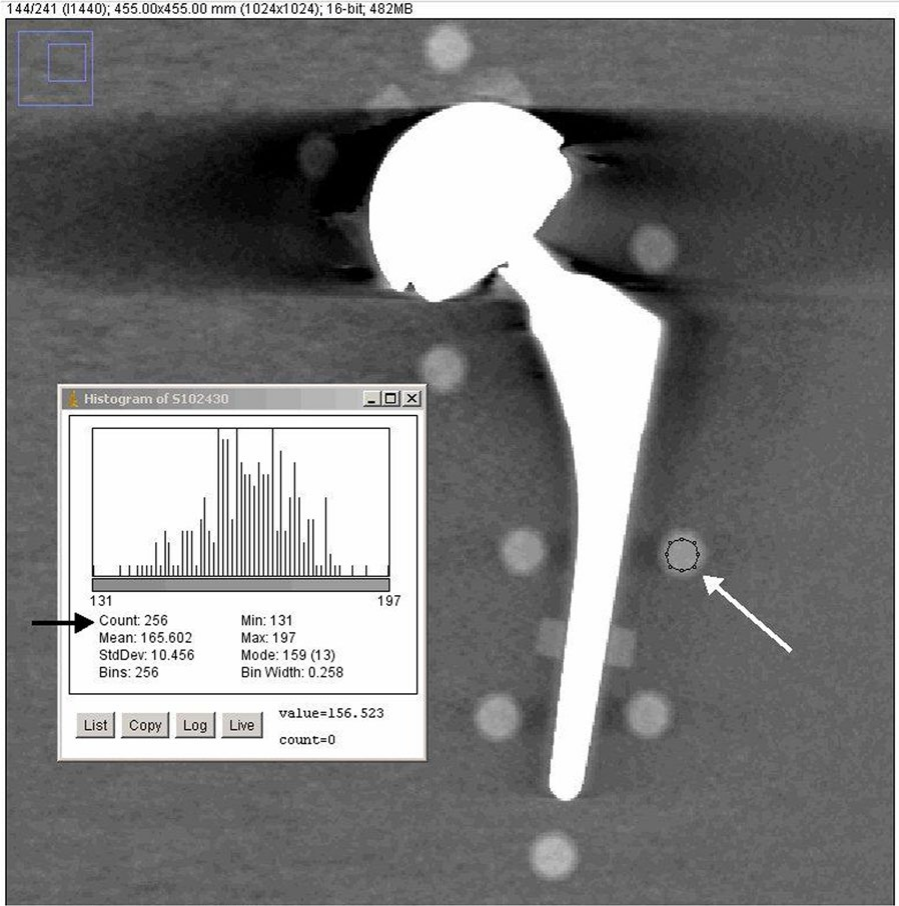
Number of pixels in ROI

The circular regions-of-interest (ROI’s) were placed over the 10 mm circular pellets, slice thickness was 1 mm. The histogram showed normal Gaussian behavior (Fig. 2). The diameter of the circle was 8 mm (c.f. 10 mm pellet size), thereby avoiding partial volume effects. The ROI’s were centrally placed on all calcium pellets together with a reference ROI in the water next to it. The used field of view of 455 mm and a matrix of 1024 × 1024 gives a pixel size of 455/1024 = 0.44 mm in the x–y plane. A circular ROI with a diameter of 8 mm this corresponds to 8/0.44 mm = 18 pixels for the diameter. Therefore in the ROI there are π(18/2)^2^ = 255 pixels, sufficient for a reliable statistical analysis, because of the high homogeneity of the pellet material. This was checked and found to be correct.

Measurements of HU values and CNRs were performed in a coronal reconstruction by using the open source software program ‘ImageJ V1.46r’ (National Institutes of Health, Bethesda, Maryland, USA). ImageJ gave the opportunity to organize the ROI placements in a template, which could be copied exactly to the same position in all the 216 acquisitions. By using this method both the phantom setup and the ROI placements were exactly the same for each acquisition. In addition, there was no change in the caudal or cranial direction, which could be the case because of the helical acquisition. ImageJ proved to accurately reproduce the ROI set on different scans and no adjustments were needed.

**Fig. 3:**
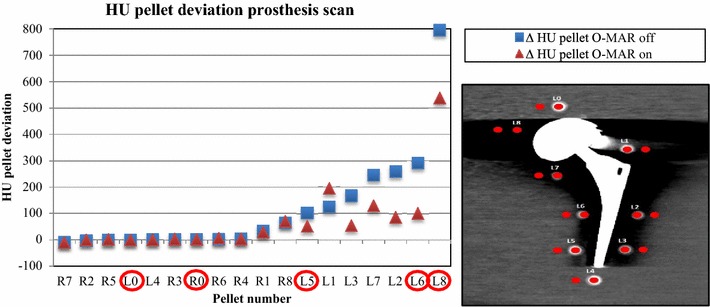
|HU| deviations compared to baseline for all pellets with large head MoM THA prosthesis in ascending order by 120 kVp, 600mAs, iDose^4^ level 5, Filter A, O-MAR on and off. The *red circled* pellets L0, R0, L5, L6 and L8 were visually chosen for degree of metal artifacts: no, light, medium and heavily disturbed

Four different pellets with different expected amounts of metal artifact influence in abovementioned positions were chosen for final analysis. Attenuation deviation reflects the difference in HU values with and without the insertion of a prosthesis. Pellet L5 was categorized as ‘light disturbance’, L6 as ‘medium disturbance’ and pellet L8 as ‘heavy disturbance’ based on visual assessment (Fig. 3). No disturbance is expected in pellet R0 and L0 due to its location in the phantom. The HU values of the pellets after prosthesis placement were compared to the corresponding pellets from the scan of the phantom without prosthesis. The effectivity of O-MAR in artefact reduction was analyzed for each of abovementioned categories of disturbance.

The HU values and SD values from the calcium pellets were used to calculate the difference between measurement and baseline. The HU values and SD values were used to calculate the CNR.

### Statistical analysis

Statistical analysis was performed by means of repeated measure ANOVA (full factorial, type ΙΙΙ), utilizing two within-subject factors for pellets (‘L0’, ‘L5’, ‘L6’, ‘L8’, ‘R0’) and O-MAR (‘off’, ‘on’), generalizing to scan protocol.

## Results

The selection of pellets for which data regarding HU values are presented in Fig. 3. It shows the deviation of the HU value for the pellets after inserting the THA in ascending order. Pellets were categorized for analysis based on the visual assessment of the degree of metal artefacts: light, medium and heavily disturbed, as can be observed in the actual scan in Fig. 3. This selection resulted in analysis of pellet L5, L6 and L8. We also analyzed L0 and R0 in order to confirm that these pellets are not disturbed by metal artefacts as could be theoretically expected, because these pellets are not in the same plane as the prosthesis. However, scatter could theoretically disturb these pellets if they were located too close to the THA.

**Fig. 4:**
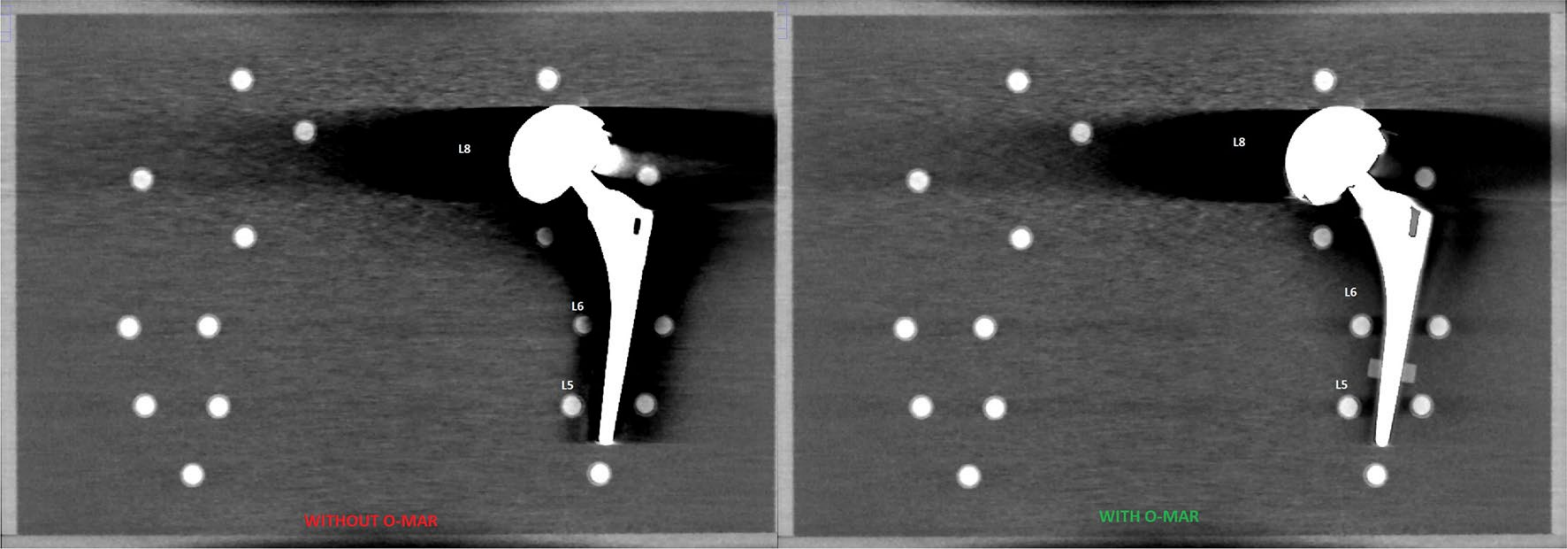
Visual observed difference of Metal artifacts caused by large head MoM THA with and without use of O-MAR, with 120 kVp, 600 mAs, filter A and iDose^4^ level 5 with a WW/WL of 360/60

**Fig. 5:**
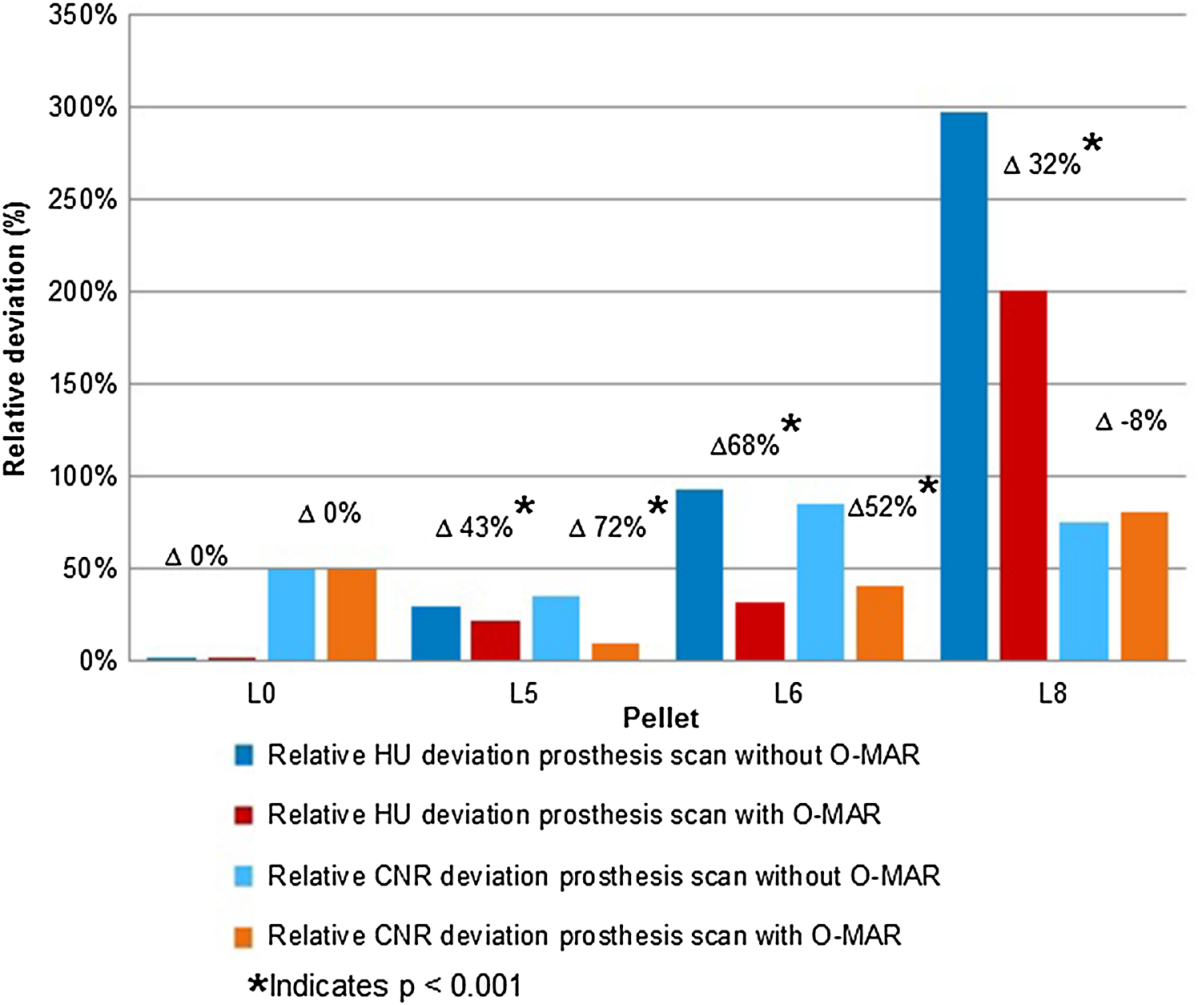
Relative HU and CNR deviation between prosthesis scans with and without O-MAR with regard to the baseline for pellets L0, L5, L6 and L8. With ∆ as relative improvement on HU deviation. Using 120 kVp, 600 mAs, filter A and iDose^4^ level 5

 Tables 2 and 3 show the absolute and relative HU deviation, respectively, and absolute CNR values in the scan with large head MoM THA prosthesis scans with and without O-MAR. These are compared to baseline for pellets L0, L5, L6 and L8 and absolute CNR values for pellets L0, L5, L6 and L8, with and without O-MAR. Figure 4 shows the scans with and without O-MAR. It can be clearly observed that the metal artefacts are reduced and subjective contrast seems improved, by looking at the better delineation of the different pellets. Figure 5 shows the relative HU and CNR deviations between prosthesis scans with and without O-MAR, with regard to the baseline for pellets L0, L5, L6 and L8. With ∆ the relative improvement on HU and CNR deviation is denoted.

**Table 2:** Absolute and relative HU deviation in the scan with large head MoM THA prosthesis scans with and without O-MAR with filter A and iDose^4^ level 5 compared to baseline for pellets L0, L5, L6 and L8

Scan protocol	Pellet	Δ |HU|	Δ HU %	Δ |HU|	Δ |HU|
		O-MAR off	O-MAR off (%)	O-MAR on	% O-MAR on (%)
120 kVp, 150 mAs	L0	0	0	0	0
	L5	102	41	52	21
	L6	291	111	101	38
	L8	795	306	538	207
120 kVp, 600 mAs	L0	0	0	0	0
	L5	79	30	57	22
	L6	238	93	82	32
	L8	766	297	517	200
140 kVp, 101 mAs	L0	6	3	6	3
	L5	115	47	70	28
	L6	276	118	81	35
	L8	796	337	531	225
140 kVp, 405 mAs	L0	0	0	13	5
	L5	94	39	57	24
	L6	271	113	83	34
	L8	799	341	517	219

Significant positive effect of OMAR (p < 0.001) for pellet L5, L6 and L6 regarding absolute and percentage of change in HU values caused by metal artefacts

**Table 3:** Absolute CNR values without and with large head MoM THA prosthesis scans with and without O-MAR for pellets L0, L5, L6 and L8

Pellet	kVp	mAs	Filter	iDose^4^ level	No MoM THA baseline	MoM THA O-MAR off	MoM THA O-MAR on
					CNR	CNR	CNR
L0	120	150	A	5	33.06	13.02	12.99
L5	120	150	A	5	22.74	13.01	19.24
L6	120	150	A	5	16.75	1.37	18.16
L8	120	150	A	5	23.12	5.18	4.19
L0	120	600	A	5	46.52	23.07	23.07
L5	120	600	A	5	41.18	26.31	37.11
L6	120	600	A	5	41.65	5.84	24.72
L8	120	600	A	5	29.71	7.32	5.74
L0	140	101	A	5	20.27	17.51	17.51
L5	140	101	A	5	18.60	8.98	14.02
L6	140	101	A	5	19.77	0.77	23.69
L8	140	101	A	5	22.94	6.61	4.64
L0	140	405	A	5	43.88	30.64	30.65
L5	140	405	A	5	27.55	19.98	30.69
L6	140	405	A	5	53.21	0.85	28.91
L8	140	405	A	5	40.34	8.51	6.13

Significant positive effect of OMAR (p < 0.001) for pellet L5 and L6 regarding absolute change in CNR values caused by metal artefacts

**Fig. 6:**
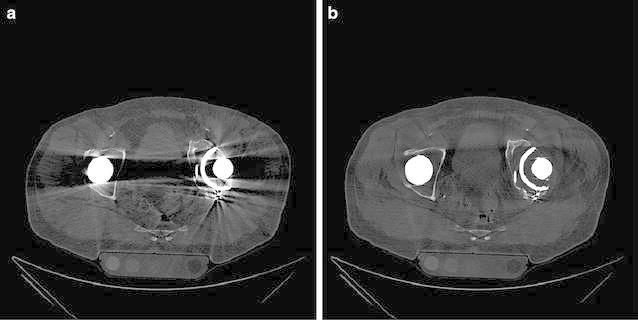
0.9 mm scans of a patient with bilateral THA; MoM THA on the *right* and conventional THA on the *left*. **a** without and **b** with the use O-MAR

To test the results for significance we used the 6 different scan protocols varying kVp and mAs, with Filter A, suitable for soft tissue, and iDose^4^ level 5, used in our current clinical practice in order to try to optimize CNR. Finally, two scans with reconstructed slice thickness of 0.9 mm are shown in Fig. 6a and b in a patient with bilateral THA, namely MoM THA on the right and conventional THA on the left, with and without O-MAR. With the use of O-MAR it is possible to view the pelvic region that was not visualized at all due to the bilateral metal artefacts.

**Fig. 7:**
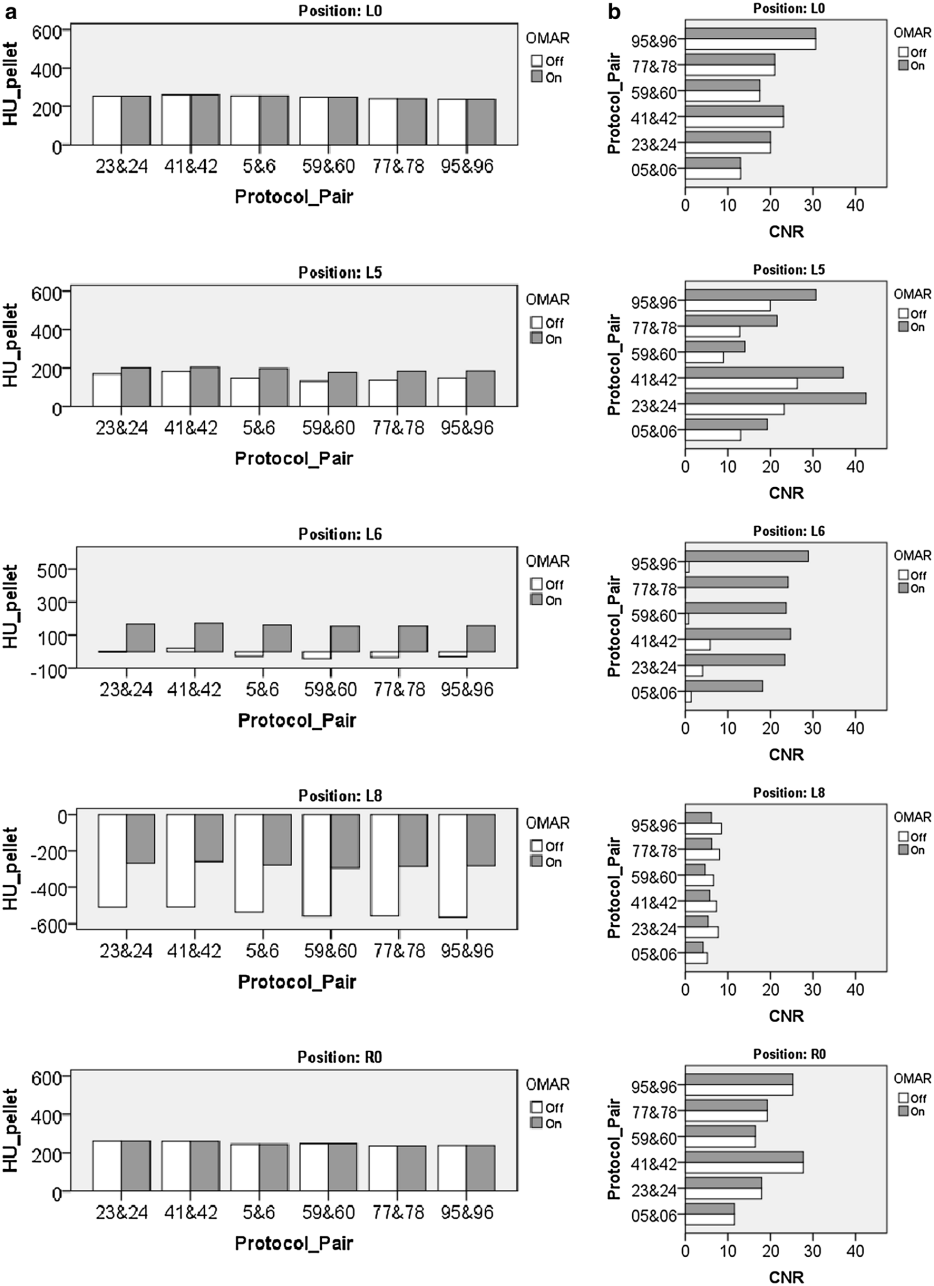
**a** HU value for each investigated pellet is shown for different scan protocols with and without OMAR. **b** CNR value for each investigated pellet is shown for different scan protocols with and without OMAR. Average beneficial significant effect for HU and CNR by use of O-MAR for each position (L0, R0 L5, L6 and L8), by six different scan settings (p < 0.001). Protocol pairs: 5 and 6, ‘120 kVp; 150 mAs; Filter A; iDose^4^ level 5’; 23 and 24, ‘120 kVp; 300 mAs; Filter A; iDose^4^ level 5’; 41 and 42, ‘120 kVp; 600 mAs; Filter A; iDose^4^ level 5’; 59 and 60, ‘140 kVp; 101 mAs; Filter A; iDose^4^ level 5’; 77 and 78, ‘140 kVp; 202 mAs; Filter A; iDose^4^ level 5’; 95 and 96, ‘140 kVp; 405 mAs; Filter A; iDose^4^ level 5’

Repeated measures ANOVA for both HU and CNR resulted in statistical significant beneficial results for pellet L5, L6 and L8 (p < 0.001) and O-MAR (p < 0.001). The interaction term for pellet and O-MAR was statistically significant as well (p < 0.001) (Fig. 7). No changes for L0 and R0 were, as expected, not found.

## Discussion

We have shown on a 64-slice CT system that the reduction of metal artefacts by O-MAR is dependent upon the disturbance caused by the metal artefact. Relative improvement in HU deviation varies between 32 and 68 %. O-MAR showed a significant improvement on CNR as well. The decrease in CNR is dependent upon the disturbance caused by the metal artifact and improvement varies from 52 and 72 %. To our knowledge, these quantitative results have not been published before with regard to large head metal-on-metal THA.

Based on literature, other researchers have investigated the effect of O-MAR in a variety of applications. Huang et al. used anthropomorphic phantoms from which they conclude O-MAR can be used in the head, thorax and is especially well suited in the pelvic area with unilateral hip prostheses. Hilgers et al. studied the CT number accuracy in large orthopedic implants in a phantom-based setting, which showed significantly better CT number accuracy in O-MAR reconstructions compared to non-O-MAR reconstructions. Li et al. evaluated O-MAR for the use of CT simulations in radiation therapy. The results indicate an improvement of the CT HU accuracy and noise, from which the authors suggest that images corrected by O-MAR are more suitable for treatment planning in radiation therapy than without. The added value of O-MAR for metal artifact reduction in CT dental applications was studied by Kidoh et al. Image noise in soft tissue corrected by O-MAR was significantly lower and O-MAR enabled imaging of structures which were not visible without O-MAR. The researchers concluded images corrected by O-MAR have a supplementary role in oral diagnosis.

All of the above study outcomes show an overall image improvement, represented by a better CT number accuracy and significant noise reduction. These findings are in line with those in this study (Hilgers et al. 2014; Huang et al. 2015; Kidoh et al. 2014; Li et al. 2012).

Despite O-MAR and showing excellent results in the reduction of metal artefacts on a 64 slice CT system, we observed that O-MAR combined with iDose^4^ level 5 was incapable of increasing CNR in heavily distorted regions (L8). We believe this is caused by photon-starvation, with associated poor photon statistics creating a spurious decrease of CNR for this particular region. The poor photon statistics in the affected region presumably lead to an inefficiency of iDose^4^ level 5 to reconstruct images with higher CNR than without O-MAR, as can be observed in low and medium affected regions. The large amount of disturbances is probably the result of a combination of both photon starvation and scatter from the large MoM THA.

The “net” effects of metal artefacts on a CT scan is a result of photon starvation, scatter and iterative reconstruction level. Model-based iterative protocols in general have shown the potential to scan with even lower dose than previous generations of iterative reconstruction (Mehta et al. 2013). It could also give the opportunity to create better images in regions with less information due to photon starvation, since the modelling might resemble the realistic situation of lowered photon statistics. Whether this can be done in combination with lower dose needs additional investigation. The effectivity of O-MAR with more novel multi-slice systems, in combination with model-based iterative reconstruction might reveal the full potential of O-MAR (Wellenberg et al. 2015).

Our phantom design was slightly oversized with a WED of 39.7 cm where a WED of 29.5 cm is representative in patients with a body-mass-index of 25. Subsequently, an elliptical phantom design instead of this rectangular phantom design could minimalize boarder artefacts. Furthermore, the use of additional pellets with different densities could give information about the depiction of soft tissue such as pseudotumours.

Follow-up patient studies must appreciate the clinical value of O-MAR in various THA populations. Improving image quality by compensating for metal artifacts by O-MAR with CT systems of a higher slice number, in combination with full iterative reconstruction will potentially enable further image quality improvements. The radiation dose regarding CT scans resulting in metal artefacts due to large metal implants such as THA, might possibly also be lowered if OMAR is applied in combination with full iterative protocols such as IMR.

## Conclusion

This phantom study shows a significant reduction of metal artefacts by O-MAR caused by MoM THA. The reduction is dependent on the amount of disturbance in attenuation caused by the metal artefact and relatively improved between 32 and 68 %. O-MAR showed a significant improvement in CNR as well. The decrease in CNR decrement is also dependent on the attenuation disturbance caused by the metal artefact, and varies between 52 and 72 %.

## Appendix

The O-MAR algorithm deploys an iterative loop where the metal sinogram is identified extracted and subsequently serves as a mask to correct the measured sinogram. The output correction image is then subtracted from the original input image. The resultant image then becomes the new input image and the process can be repeated. The first step is to establish a threshold in the input image to create a metal-only image. The metal-only image consists of all pixels set to zero except for those pixels categorized as metal. The metal data points in the sinogram are replaced with interpolated values, which will simulate tissue in place of the metal. This sinogram is back projected and the resultant image is used to segment tissue and create the tissue-classified image. For subsequent iterations, this step is not performed. After one or several rounds, if no large clusters of metal pixels are present in the image, no further processing is performed and the final, corrected images are calculated.
